# Re-Engineering the Human Resource Strategies Amid and Post-Pandemic Crisis: Probing into the Moderated Mediation Model of the High-Performance Work Practices and Employee's Outcomes

**DOI:** 10.3389/fpsyg.2021.710266

**Published:** 2021-07-09

**Authors:** Ma Zhiqiang, Hira Salah ud din Khan, Muhammad Salman Chughtai, Li Mingxing

**Affiliations:** ^1^School of Management, Jiangsu University, Zhenjiang, China; ^2^Faculty of Management Sciences, International Islamic University, Islamabad, Pakistan

**Keywords:** high-performance work practices, organization-based self-esteem, positive psychological capital, employee's in-role performance, employee's task performance, COVID-19 pandemic

## Abstract

By incorporating the conservation of resource theory, this study examines how high-performance work practices (HPWPs) affect the employee's in-role performance (EIRP) and employee's task performance (ETP) during the coronavirus disease 2019 (COVID-19) pandemic. Furthermore, this study investigates how organization-based self-esteem (OBSE) and positive psychological capital (PPC) affect the relationship between HPWPs and outcomes of employees such as EIRP and ETP. A quantitative technique based on the survey method was used to gather the primary data of the investigation. Two hundred and eleven bank employees working in different banks of Punjab and Pakistan participated in the survey process. The PROCESS-macro was used to test the relationship among the study constructs. Our results supported all the study hypotheses, however positive psychological capital did not moderate the indirect effect of high performance work practices on employee's task performance via organization based self-esteem. This study is the earliest of its kind that focuses on HPWPs and outcomes of employees amid the COVID-19 pandemic in a developing country like Pakistan. The findings of this study provide practical implications in the post and continuing pandemic situation for organizations to human resource management to redesign workforce strategies to increase their contribution and responses to realize organizational priorities. Although researchers have explored the topic in different sectors, scant studies have investigated the potential impact, barriers, and enabling mechanisms that function as a catalyst in HPWPs during the pandemic situation.

## Introduction

Novel coronavirus disease 2019 (COVID-19) epidemic broke out in China (Wuhan) in December 2019 (Zhu et al., 2020), which was declared a pandemic by WHO in January 2020. It spread increasingly all over the world and hit Pakistan in March 2020. This worldwide outbreak led countries to lockdown everything to control the possible spread of fatal diseases. These drastic measures affected the major sectors of the economy, which are considered the economy of the lifeblood of every country, for instance, financial institutions (Disemadi et al., 2020). During this pandemic, the workforce of these institutions had to perform their jobs despite the threat of catching the contagious virus (Zhu et al., 2020). The employees of these organizations are at high risk as this virus is present on currency notes, checks, and demand drafts, which survive longer than a common virus. This situation leads to physical illnesses and affects the psychological health of employees (Zhu et al., 2020), especially in financial institutions. These circumstances have adversely influenced the developing countries leading to massive economic crises due to increased downsizing by the private sector organizations; thus, it developed uncertainty and job insecurity giving rise to intolerant and depressive behaviors (Godinic et al., 2020). They lack appropriate resources, such as practical skills, strategies, and policies, that boost the morale of employees (Alatailat et al., 2019; Diogo and Da Costa, 2019; Kumar et al., 2020), essential for high performance in work.

The current scenario of COVID-19 has drawn the focus of the organizations to reengineer strategies in high-risk environments (Kumar and Reddy, 2019). The financial institutions (i.e., banking sector) especially need to develop and implement such human resource policies and performances that enable employees to perform their roles and tasks efficiently in an unfavorable atmosphere (Al-Dalahmeh et al., 2018; Aeknarajindawat et al., 2020). Effective policies that cover the overall management of the resources, especially human resources, bring productivity, high performance, and organizational success (Obeidat et al., 2016).

This study sheds light on the utmost important role of high-performance work practices (HPWPs) by employing the conservation of resource (COR) theory in organizations to help achieve organizational objectives. COR (Hobfoll, 1989) theory suggests that resources (i.e., physical, psychological, organizational, and emotional) are the significant aspects of well-being and satisfaction and are beneficial for individuals to gain more resources (Hobfoll, 1989). Studies indicated that HPWPs are associated with motivation, resilience, self-assurance, and confidence of an individual, resulting in positive outcomes of employees (Alatailat et al., 2019; Arefin et al., 2019; Diogo and Da Costa, 2019; Ismail et al., 2021). HPWPs through their interaction between organization and employees rise the mutually beneficial impact (Boon et al., 2019) that resultantly increases the positive outcomes and reduces the adverse ones. Organizations use HPWPs as an imperative management tool for maximization of the performance of individuals (Karatepe and Olugbade, 2016), which not only increase the profitability of the firm but also increase the intellectual capital level of the organization through the enhancement of competencies (Boon et al., 2019). Achievement of competitive advantage and strength is possible through the development of human capital of the organization (Khan et al., 2019b); for that purpose and for the optimization of the performance of individuals, organizations adopt HPWPs (a list of human-related policies and practices), e.g., job design, extensive training and development, attractive reward and compensation system, and information sharing (Alatailat et al., 2019; Zhang et al., 2019; Rubel et al., 2020).

However, few studies have focused on how organizations could incorporate and introduce HPWPs in the banking industry (Huo and Boxall, 2018; Cooper et al., 2019) and its potential impact on the outcomes of employees, i.e., employee's in-role performance (EIRP) and employee's task performance (ETP).

Accordingly, this study focused on the supportive organizational mechanisms, such as organization-based self-esteem (OBSE), which can serve as a mediator between HPWPs and EIRP and ETP. Scholars characterized OBSE as the extent to which the workforce perceives that they are appreciated and valued by the organization (Pierce et al., 1989). HPWPs are employee-oriented strategies that improve individual skills (Chughtai, 2017; Zhang et al., 2018; Diogo and Da Costa, 2019). Given this, OBSE is an element that cultivates motivation and self-confidence that further translates into extra EIRP and ETP (Pierce et al., 1989; Yang et al., 2019). Therefore, in the circumstances of the COVID-19 pandemic, employees need organizational support to reduce the stress; if organizations extend their support in the form of self-esteem, the EIRP and ETP increase.

Furthermore, this study tries to understand the positive psychology approach by pinpointing the positive psychological capital (PPC) that acts as a personal resource for the individuals, enabling them in a challenging working environment (Luthans et al., 2007). Scholars ordained that PPC, also known as a workforce positive emotional condition, allows personal development (Luthans et al., 2007; Luthans and Youssef-Morgan, 2017). Past studies revealed that PPC positively affects work engagement, job performance, attitudes, and behaviors (Luthans et al., 2007; Luthans and Youssef-Morgan, 2017; Kotzé, 2018). Based on this, PPC could help meet the challenging environment for employees amid COVID 19, since the pandemic has affected the well-being of employees and drained their energies to perform efficiently (Mao et al., 2020). Thus, we proposed that PPC can act as a moderating construct between HPWPs and OBSE relationship and the outcomes of employees (i.e., EIRP and ETP) in the COVID-19 epidemic situation (Kim, 2020). This study attempts to answer the following questions: “How do HPWPs influence the performance-related outcomes of employees?” and “How do OBSE and PPC influence the HPWPs and link outcomes of employees amid and post-pandemic times?”

**Figure 1:**
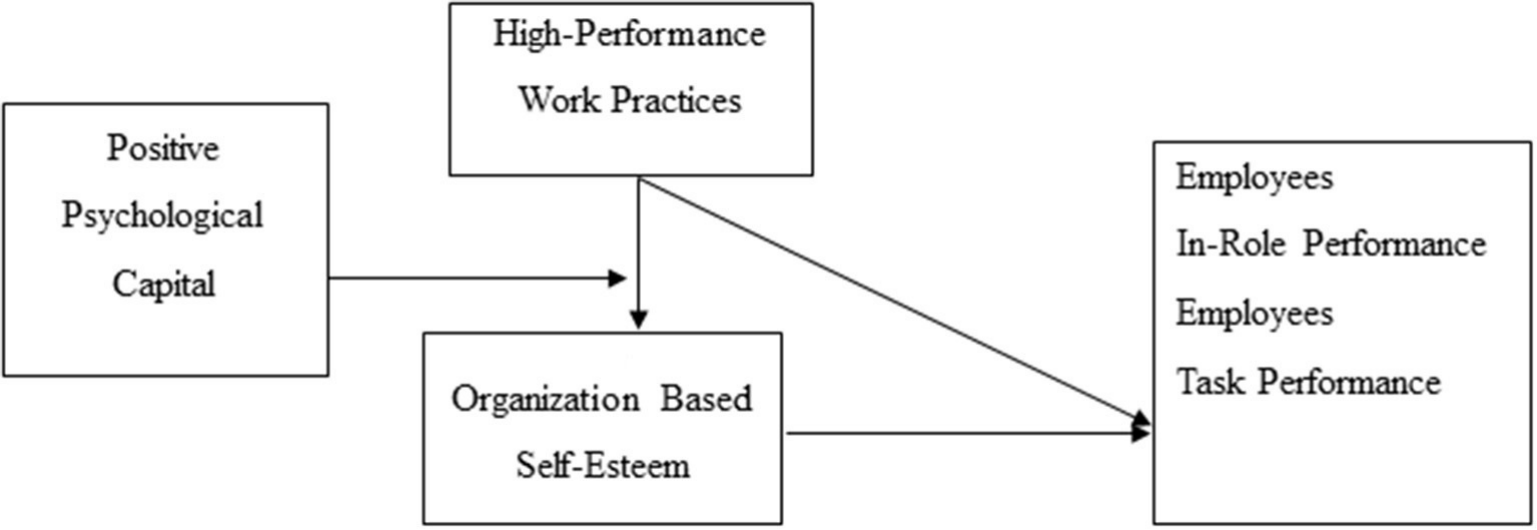
Conceptual model.

The objectives and manifold contribution of this study are as follows: first, this study investigates the direct influence of HPWPs on the outcomes of employees (i.e., EIRP and ETP). Second, we explored the mediating role of OBSE in the link between HPWPs and outcomes of employees (i.e., EIRP and ETP). Third, this study examined the moderating effect of PPC on the link between HPWPs and outcomes of employees. Fourth, the moderated mediation impact of PPC and OBSE was tested in the link between HPWPs and outcomes of employees. Additionally, in this study, we overcame the gap suggested by Gahan et al. (2020), Nasurdin et al. (2020), and Rubel et al. (2020), as these authors advanced to use the supervisor-subordinate and self-assessed data for the validation of the outcomes and to explore under which circumstance HPWPs produce better performance. Additionally, this study also responds to the studies by Han et al. (2020), Iyanda Ismail et al. (2020), and Nasurdin et al. (2020), where they suggested to explore the influence of HPWPs on psychological capital (Okun, 2020) in other organizational setups with different outcomes of employees. Finally, employing COR theory adds to the literature of organizational behavior by examining the current framework field in the developing country, i.e., Pakistan, particularly in the COVID-19 pandemic, as shown in Figure 1.

## Theoretical Foundation

Conservation of resource theory provides the foundation for the support of this study model that establishes that resources are the ideal set of skills helping employees to accomplish workplace tasks (Hobfoll, 1989; Hobfoll et al., 2018). At present, in high work demand conditions as faced by organizations globally due to the COVID-19 epidemic, employees need to develop self-protective strategies to protect their existing resources (Hobfoll et al., 2018). Finally, scholars explained that instituting HPWPs culture in organizations enables the workforce to develop skills aligned with the organizational goals and strategies (MacDuffie, 1995). Empirical studies have suggested that implementing HPWPs in the organizational arena (Kim and Liu, 2017; Iqbal, 2019; Kumar and Reddy, 2019; Nansubuga et al., 2019; Ogbonnaya and Messersmith, 2019) leads to different positive consequences such as increased well-being, knowledge sharing behavior (Ali et al., 2021), and high individual and organizational performance (Khan et al., 2021). Furthermore, HPWPs enable employees to gain resources that increase EIRP and ETP through ability, motivation, and participation at the workplace.

According to the COR theory, social, physical, supportive environmental, and cognitive resources are the significant resources that helps to protect and enhance well-being (Hobfoll, 1989; Hobfoll et al., 2018), also become valuable, especially in the current scenario of the COVID-19 pandemic. Given this, we argued that OBSE acts as a conduit in a psychological and organizational resource that spurs self-determination and value for the entity (Pierce et al., 1989). OBSE is also an essential occupational resource that aids in positive outcomes (Hobfoll et al., 2018). Following this, employees are likely to demonstrate more work activities and put extra effort in performing their duties efficiently, especially in uncertain circumstances.

Additionally, personal resources empower individuals to manage and cope with environmental stressors (Hobfoll, 1989; Hobfoll et al., 2018; Khan et al., 2019a). Based on this, PPC is a beneficial cognitive resource that demonstrates the characteristics of dealing with occupational demands (Siu et al., 2014), especially in the environment of uncertainty caused by the pandemic. Researchers elaborated that PPC is a collection of positive elements that include resilience, self-efficacy, optimism, and hope, reducing the adverse outcomes (Luthans et al., 2007; Kim and Beehr, 2018b). Concerning the disruptive COVID-19 pandemic, the major stress-causing factor is the absence of positive resources that led employees to feel demotivated (Kim, 2020). Furthermore, through COR theory, we unveiled the importance of specific resources that protect employees from emotional loss (Hobfoll et al., 2018), which employees face in accomplishing their professional responsibilities (i.e., EIRP and ETP).

### High-Performance Work Practices, EIRP, and ETP

The burgeoning literature advocated that organizations following HPWPs indicated improved motivation, high-quality services, products, increased innovative task behaviors, creativity, job crafting, efficiency, low levels of absenteeism, and turnover of employees (Kim et al., 2015; Messersmith et al., 2018; Ali et al., 2019; Chughtai and Rizvi, 2019; Aeknarajindawat et al., 2020; He et al., 2021; Li et al., 2021). Additionally, HPWPs augment the skills, capabilities, and intellectual capital of employees, especially in service industries (Ali et al., 2018; Rehman et al., 2020). Recent studies revealed the influential role of HPWPs in human resource management strategies in Western culture; however, limited studies have explored its impact on developing economies (Cooke, 2018; Cooke et al., 2019). Past literature emphasized the potential effect of HPWPs on task performance (Topcic et al., 2016; Cooper et al., 2019; Jeong and Shin, 2019; Yang et al., 2019). Furthermore, researchers conceded a need to explore how HPWPs facilitate extra roles and performances of employees (Karadas and Karatepe, 2019). Thus, this study focused on the impact of HPWPs as recommended by researchers (Chamberlin et al., 2018; Huang et al., 2018; Murphy et al., 2018; Pak and Kim, 2018). Therefore, we investigated the link between HPWPs and performance-related outcomes of employees amid the COVID-19 situation. Thus, it is postulated that

**Hypothesis 1a** *HPWPs positively affect EIRP*.

**Hypothesis 1b** *HPWPs positively affect ETP*.

### Organization-Based Self-Esteem as Mediator

Researchers characterized OBSE as a mechanism that helps employees realize their personal and organizational goals (Pierce et al., 1989). Scholars expressed that organizational support increases the level of self-esteem of employees and improves their behaviors toward their jobs (Tetteh et al., 2019). Self-esteem represents the self-concept of individuals, which plays a vital role in the lives of everyone, affecting psychological, physical well-being, enthusiasm, and life satisfaction (Cameron and Granger, 2019). In addition, earlier studies revealed that OBSE engenders many positive outcomes, i.e., stress management (Costantini et al., 2019), individual performance (Hahn and Mathews, 2018) and organizational citizenship behaviors (Kim and Beehr, 2018a), and innovative behaviors (Ali, 2021; Wen et al., 2021). Moreover, OBSE reduces uncertainty (Neves et al., 2020), as individuals having OBSE can handle stressful situations effectively (Costantini et al., 2019). Further studies on OBSE concerning the implementation of HPWPs can be favorable for organizations that are looking to have improved performance (Zheng et al., 2019; Carrion, 2020). Based on the above discussion, this study attempts to investigate the mediating role of OBSE in the link between HPWPs and EIRP and ETP. Thus, it is postulated that

**Hypothesis 2a** *OBSE mediates the relationship between HPWPs and EIRP*.

**Hypothesis 2b** *OBSE mediates the relationship between HPWPs and ETP*.

### Positive Psychological Capital as a Moderator

Positive psychological capital is defined as “positive psychological state of development of an individual which is characterized by (1) having confidence (self-efficacy) to take on and put in the necessary effort to succeed at challenging tasks, (2) making a positive attribution (optimism) about succeeding at present and in the future, (3) persevering toward goals and, when necessary, redirecting paths to goals (hope) to succeed, and (4) when beset by problems and adversity, sustaining and bouncing back and even beyond (resilience) to attain success” (Luthans et al., 2007). Accordingly, the banking industry has to deal with highly challenging work conditions that give rise to a stressful atmosphere to meet organizational competitiveness (Goetz et al., 2019). In the scenario of the COVID-19 pandemic, the performance of financial institutions is adversely affected (Disemadi et al., 2020), and the workforce of these institutions suffers from stress, anxiety, and psychological and emotional loss (Sembiring et al., 2020). Additionally, scholars expressed that PPC increases interpersonal citizenship behaviors (Khliefat et al., 2021) and innovative behaviors (Mutonyi, 2021) and decreases job insecurity (Wang et al., 2021). Given this, employees who possess high PPC can perform any demanding tasks and are ready to adjust to new practices and procedures. With this viewpoint, it can be anticipated that HPWPs stimulate the confidence of employees in an organization and result in increased OBSE when PPC is high. Thus, it is hypothesized that

**Hypothesis 3** *PPC moderates the positive relationship between HPWPs and OBSE*.

### An Integrative Moderated Mediation Model

This integrative model hypothesized that HPWPs could promote EIRP and ETP (Hypothesis 1a and 1b). This study also proposes that the influence of HPWPs increases extra role performances of employees indirectly through OBSE (Hypothesis 2a and 2b). Moreover, it is anticipated that the strength of the link between HPWPs and OBSE would be contingent on the PPC level (Hypothesis 3). Additionally, based on the above discussion, we proposed a moderated mediation framework in which PPC moderates the indirect effect of HPWPs on EIRP and ETP *via* OBSE. Thus, it is hypothesized,

**Hypothesis 4a** *PPC moderates the indirect positive effect of HPWPs and EIRP via OBSE in the sense that a higher level of PPC will strengthen the indirect impact of HPWPs*.

**Hypothesis 4b** *PPC moderates the indirect positive effect of HPWPs and ETP via OBSE in the sense that a higher level of PPC will strengthen the indirect impact of HPWPs*.

## Materials and Methods

### Sample and Procedures

This study targeted the banking sector due to the ongoing economic crisis (amid pandemic) that has globally affected human and financial resources. It is imperative to study the factors that help understand and realign the strategies to improve the banking sector workforce performance in Pakistan. The data were collected through social contacts with bank staff and random personal visits of public and private banks. Each survey was attached with a cover letter to elucidate the aim of this study to the respondents and ensure confidentiality. The temporal separation method was adopted in this study to overcome the issues related to common method bias (Podsakoff et al., 2012). The 1-month temporal separation method was used in this study as suggested by (Podsakoff et al., 2012). In the first lag, the data were collected for independent and mediating variables from employees. In the second lag, we invited employees to fill the survey regarding the moderating variable. The dependent variable questionnaires were filled by the immediate officers of those employees who participated at the first lag. All questionnaires were marked with specific codes of identification to match the surveys of both time periods. A total of 400 questionnaires were distributed through self-administered data collection method and 290 questionnaires received at the end of the first lag, which were correctly filled; at the end of the second lag, 211 responses were finalized for further analysis that constituted a response rate of 52.75%.

### Measures

All the measures of this study were assessed on a 5-point Likert scale starting from 1 (“strongly disagree”) to 5 (“strongly agree”). The measures used for this study are as follows: ***HPWPs:***this scale comprised of 10 items developed by Sun et al. (2007) was used that indicated the reliability of 0.92. ***OBSE:***10-item scale developed by Pierce et al. (1989) was adopted to measure OBSE, showing the reliability of 0.94. ***PPC:***this scale was assessed by Luthans et al. (2007), which comprises 10 items to measure PPC that demonstrated the reliability of 0.94. ***EIRP*:** the 7-item scale was used, which was developed by Williams and Anderson (1991). The Cronbach's alpha indicated a value of 0.94. ***ETP:***the 7-item scale developed by Koopmans et al. (2014) was used, indicating the reliability of 0.93.

## Results

### Analysis Strategy

We used PROCESS-macro to test the relationship between the study variables. It was employed to test the complex moderated mediation as suggested by Hayes (2018). Additionally, it enables testing the complex relationship among the variables related to organizational behavior studies (Hayes, 2018).

### Demographics

Most of the participants were males, i.e., 148 (70.1%), whereas 64 (29.9%) were females. The age distribution indicated that 111 (52.6%) participants were between 20 and 30 years, 92 (43.6%) were between 31 and 40 years, and 8 (3.8%) were between 41 and 50 years. Educational qualification showed that 29 (13.7%) participants had MS/MPhil, 135 (64.0%) master's degree, and 47 (22.3%) had a graduate degree. The information of work experience consisted of 43 (20.4%) participants having “ <1 year,” 105 (49.8%) having “1–5 years,” 49 (23.2%) having “6–10 years,” 12 (5.7%) having “11–15 years,” and 2 (1.0%) having “21 years above.”

### Descriptive Statistics and Correlations

Table 1 presents the correlation coefficients and a descriptive statistic of the constructs. The results demonstrated that all variables of this study were positively correlated with each other at the significance level of 0.01.

**Table 1:** Correlations, descriptive, and reliability statistics.

		**Mean**	**SD**	**α**	**1**	**2**	**3**	**4**	**5**
1	HPWPs	3.74	0.8257	0.92		0.301^**^	0.422^**^	0.470^**^	0.305^**^
2	OBSE	4.01	0.8139	0.94			0.480^**^	0.478^**^	0.314^**^
3	PPC	3.85	0.8542	0.94				0.502^**^	0.315^**^
4	EIRP	4.05	0.6713	0.94					0.384^**^
5	ETP	4.06	0.6158	0.93					

*HPWPs, high-performance work practices; EIRP, employee's in-role performance; ETP, employee's task performance; OBSE, organizational based self-esteem; PPC, positive psychological capital; SD, standard deviation; α, reliabilities*.

** *p < 0.01*.

### Hypotheses Testing

Table 2 indicated that there was a positive influence of HPWPs on EIRP (where *b* = 0.20, *t* = 3.61, and *p* < 0.001) and a positive impact on ETP (where *b* = 0.11, *t* = 2.47, and *p* < 0.001). Further bootstrapping has also been conducted to test the indirect effects. By using PROCESS-macro, we computed a 95% CI through 5,000 samples for each mediation effect as suggested by Hayes (2018). The indirect impact of HPWPs on EIRP *via* OBSE was significant as 95% CI did not contain zero and *b* = 0.18 (95% CI ranging from 0.05 to 0.33); thus, H2a received further support. Similarly, the indirect impact of HPWPs on ETP *via* OBSE is insignificant as 95% CI did not contain zero and *b* = 0.12 (95% CI ranging from 0.01 to 0.25); thus, H2b also found further support.

**Table 2:** Direct and indirect effect results (both dependent variables).

**Model**	**HPWPs** **→** **OBSE** **→** **EIRP**			
	**b**	**SE**	**LL**	**UL**
Direct Effect (Bootstrap)	0.20	0.05	0.09	0.30
Indirect Effect (Bootstrap)	0.18	0.07	0.05	0.33
**Model**	**HPWPs** **→** **OBSE** **→** **ETP**			
	**b**	**SE**	**LL**	**UL**
Direct Effect (Bootstrap)	0.11	0.05	0.02	0.2
Indirect Effect (Bootstrap)	0.12	0.07	0.01	0.25

*HPWPs, high-performance work practices; EIRP, employee's in-role performance; ETP, employee's task performance; OBSE, organizational based self-esteem; PPC, positive psychological capital; bootstrap sample size = 5,000; SE, standard error; LL, lower limit; UL, upper limit*.

### Moderation Analysis

**Figure 2:**
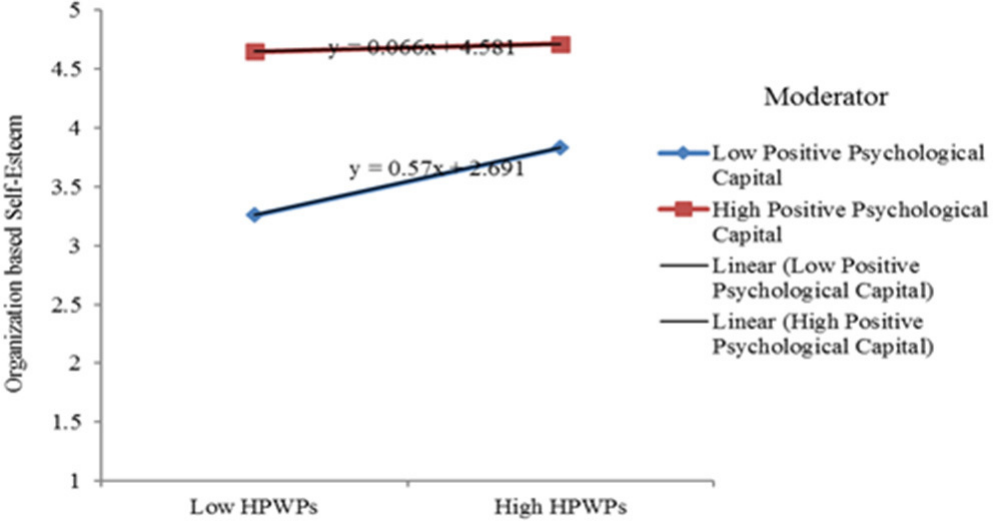
Interaction effects.

Furthermore, the moderating effect of PPC has also been tested between the link of HPWPs and OBSE (mediating variable). As shown in Table 3, the interaction of HPWPs and PPC was significantly related to OBSE (*b* = 0.12 and *p* < 0.001), which provides support for H3. Moreover, we also plotted the slopes for the interaction effect (HPWPs × PPC) on OBSE, as shown in Figure 2, which revealed that a higher level of PPC with a higher level of HPWPs increases the OBSE of employees.

**Table 3:** Moderation Results.

**Variable**			**Organization based Self-Esteem**		
	**b**	**SE**	***t*-value**	***p*-value**	**LL/UL**
Intercept	4.09***	0.02	210.46	0.00	4.05/4.12
HPWPs	0.15***	05	3.30	0.00	0.06/0.25
PPC	0.57***	0.04	12.89	0.00	0.48/0.66
HPWPs x PPC	0.12***	0.01	9.72	0.00	0.15/0.10
*R^2^ Change*	0.03				
*F*	94.54***				

*HPWPs, high-performance work practices; EIRP, employee's in-role performance; ETP, employee's task performance; OBSE, organizational based self-esteem; PPC, positive psychological capital; SE, standard error; LL, lower limit; UL, upper limit*.

### Moderated Mediation Analysis

This study also examined the conditional indirect effects using the PROCESS Model-07 (Hayes, 2018). Furthermore, Table 4 demonstrates that PPC moderates the indirect effect of HWPSs on EIRP *via* OBSE; it is significant for the high/low level of PPC because it does not contain zero. Moreover, Table 4 demonstrates that PPC did not moderate the indirect effect of HPWPs on ETP *via* OBSE because it was not found significant for the high/low level of PPC, as zero was found between the upper and lower level CI. Moderated mediation index, as depicted in Table 4, showed *b* = 0.03, boot SE = 0.01, and LL (lower limit)/UL (upper limit) = 0.05/0.01; thus, H4a was supported. This result indicated that PPC moderated the indirect effect of HPWPs on EIRP *via* OBSE in such a way that the higher the level of PPC will shape, the higher the level of EIRP. Additionally, Table 4 depicted *b* = 0.02, boot SE = 0.01, and LL/UL = 0.04/0.00); thus, H4b did not get support. This result indicated that PPC did not moderate the indirect effect of HPWPs on EIRP *via* OBSE.

**Table 4:** Conditional indirect effects (PPC as moderator).

**Mediator (EIRP as *Dependent Variable*)**	**PPC**	**Effect**	**Boot SE**	**Boot LL and UL**	
OBSE	(−1 SD)	−0.85	0.06	0.03	0.02,0.13
OBSE	(+1 SD)	0.85	0.01	0.01	0.01,0.05
**Mediator (ETP as** ***Dependent Variable*****)**	**PPC**	**Effect**	**Boot SE**	**Boot LL and UL**	
OBSE	(−1 SD)	−0.85	0.04	0.02	0.00,0.09
OBSE	(+1 SD)	0.85	0.01	0.02	0.00,0.04
**Moderated Mediation Index**					
**Mediator (PPC as Moderator)**	**b**	**Boot SE**	**Boot LL and UL**		
OBSE (*EIRP-Dependent Variable*)	0.03	0.01	0.05,0.01		
OBSE (*ETP-Dependent Variable*)	0.02	0.01	0.04,0.00		

*PPC, positive psychological capital; HPWPs, high-performance work practices; OBSE, organizational based self-esteem; EIRP, employee's in-role performance; ETP, employee's task performance*.

## Discussion

This study investigated the impact of HPWPs concerning performance-related outcomes of employees with mediating impact of OBSE and moderating impact of PPC. Furthermore, this study also examined the moderated mediation link between the variables in the banking industry of Pakistan amid the COVID-19 pandemic.

Generally, the results of this study provided support for most of our proposed hypotheses. The findings of our first hypothesis that proposed HPWPs positively influencing the outcomes of employees were supported and consistent with earlier studies (Edgar et al., 2019). The results suggest that performance-related measures of employees are significant indicators of HPWPs. The second hypothesis that postulated the positive indirect influence of OBSE between the relationship of HPWPs and outcomes of employees was also supported. The findings of this hypothesis were in line with the findings of Yang et al. (2019). Furthermore, COR theory also supported that vital resources produce a positive impact on the work outcomes of employees. Moreover, this finding established that OBSE is an essential source of internal motivation associated with workforce attitudinal and behavioral consequences (Pierce et al., 1989). The next hypothesis regarding the moderating role of PPC on the link between HPWPs and OBSE was also found significant and supported by previous studies (Aybas and Acar, 2017). The results showed that PPC has a substantial moderating impact on HPWPs and OBSE relationships. Thus, high PPC strengthens the association between HPWPs and OBSE, which is evident in the pandemic situation that PPC provides psychological support to employees that positively influence the perception of self-esteem about their organizations. Our final hypothesis concerning the moderated mediation model with both dependent variables (i.e., EIRP and ETP) demonstrated that PPC moderates the indirect effect of HPWPs on EIRP *via* OBSE. In other words, a higher level of PPC with OBSE provides psychological resources to employees to perform better EIRP in the presence of HPWPs, especially in stressful circumstances, e.g., COVID-19. Contrarily, PPC did not moderate the indirect effect of HPWPs on ETP *via* OBSE, which revealed that some factors affected the motivation of employees to perform their tasks efficiently. Subsequently, the COR theory elaborated that resource losses in the work environment have more significant influence than appreciated gains. Thus, we approbated that due to some resource loss, the ETP was not improved as it was anticipated.

### Theoretical Contributions

The findings of this study contribute to the literature on organizational behavior and human resource management in many ways. First, the results indicated a significant and positive link between HPWPs and performance-related outcomes of employees. Although previous studies focused on the relationship between HPWPs and organizational performance, its effect on performance-related measures of employees was not investigated. The positive impact of HPWPs on EIRP and ETP demonstrated that organizational investment in human resource practices is essential to gain, sustain, and improve the skills of employees to enhance productivity and efficiency.

Furthermore, this study extended by investigating the mediating role of OBSE in the link between HPWPs and employee performance. OBSE contributed to spurring HPWPs at the workplace, which eventually affects the EIRP and ETP. As the pandemic situation has caused high stress, low morale, and general well-being, we revealed that OBSE acts as a vital psychological resource nurtured by the organizations to their workforce to accomplish their goals. Moreover, this study contributed by exploring the moderating role of PPC on the link between HPWPs and OBSE. Consequently, PPC proved to be an essential psychological resource that results in high productivity and efficiency, such as EIRP and ETP. Additionally, the moderated mediation link was unveiled between the variables that demonstrated how vital resources (i.e., OBSE and PPC) aid in achieving and cultivating positive employee performance.

Our holistic model in the current pandemic scenario presents a unique model that further helps scholars carry out future studies. Finally, we examined the study variables and their relationships in a non-Western context in Pakistan. Also, we investigated banking sector employees in pandemic situations where resource loss is an inevitable element. To overcome this situation, the impact of necessary personal/organizational resources was explored that helps employees perform better.

### Practical Implications

Our findings present numerous implications for the researchers, practitioners, and management of different organizations on how to enhance the performance of employees amid and post-pandemic situations. As scholars, Hobfoll et al. (2018) advocated that organizations intend to increase their chance of meeting their goals and they must create an atmosphere loaded with resources that empower the workforce to grow (Khan et al., 2017). Our findings are in line with this standpoint by signifying that HPWPs are associated with psychological resources of employees related to performance (Luthans et al., 2007; Hobfoll et al., 2018). Executives and higher management can create an atmosphere that increases self-esteem of individuals and values their participation. The organizations could arrange workshops to train their workforce to develop personal skills such as PPC. This resource increases the motivation of employees to work efficiently in critical times. Policymakers of the organizations must pay attention to the physical and psychological health of their workforce through OBSE and by enhancing their level of psychological capital, which increase their efficacy level and resulted in higher outcomes (Ali et al., 2020) especially in the post-pandemic situation that requires a strong psychological state of workforce.

### Limitations and Future Directions

The limitations of this study are as follows: first, this study focused on the banking industry; the findings may not be generalized in other occupational areas like manufacturing, service sectors such as textiles, telecommunication, and public sector organizations in Pakistan. Thus, future studies can be carried out in other sectors of the economy. Second, we collected data from a developing economy; future studies replicate our findings in another cultural context to examine the influence of cultural settings. Third, we used OBSE as the mediator and PPC as the moderator; future researchers are encouraged to examine some other moderators and mediators between HPWPs and outcomes of employees, for instance, personal initiative, leadership, and perceived organizational support from other theoretical viewpoints.

## Data Availability Statement

The raw data supporting the conclusions of this article will be made available by the authors, without undue reservation.

## Ethics Statement

The studies involving human participants were reviewed and approved by Faculty of Management Sciences, International Islamic University, Islamabad, Pakistan. The patients/participants provided their written informed consent to participate in this study.

## Author Contributions

MZ and HK: definition of research objectives, models, and hypotheses. MC: the provision of materials (i.e., questionnaires) and data collection. LM: article revision and proofreading. All the authors contributed and approved the final draft for publication.

## Conflict of Interest

The authors declare that the research was conducted in the absence of any commercial or financial relationships that could be construed as a potential conflict of interest.
